# Potentially traumatic childbirth experience, childbirth-related post-traumatic stress disorder symptoms, and the parent-infant relationship in non-birthing parents

**DOI:** 10.1186/s12884-025-07200-3

**Published:** 2025-02-04

**Authors:** Rebecca Hunter, Leonardo De Pascalis, Kieran Anders, Pauline Slade

**Affiliations:** 1 Department of Primary Care and Mental Health, University of Liverpool, Bedford Street South, Liverpool, L69 7ZA UK; 2 Department of Psychological Science, University of Liverpool, Bedford Street South, Liverpool, L69 7ZA UK; 3 Department of Psychology, University of Bologna, Viale Berti Pichat 5, Bologna, 40127 Italy; 4 Home-Start HOST, Ryecroft Hall, Manchester Road, Audenshaw, M34 5ZJ UK

**Keywords:** Birth trauma, Non-birthing parents, Parent-infant relationship, CB-PTSD, Childbirth

## Abstract

**Background:**

Non-birthing parents are typically present for the birth of their infants. Evidence suggests that some non-birthing parents may experience witnessing childbirth as traumatic, with some going on to develop childbirth-related post-traumatic stress disorder (CB-PTSD). This study aimed to explore the associations between non-birthing parents’ experiences of childbirth, symptoms of CB-PTSD, and the parent-infant relationship. The COVID-19 pandemic context is considered throughout the study, although it must be noted that most data were not collected during UK lockdown restrictions.

**Methods:**

A cross-sectional design was utilised. Participants were non-birthing parents who were present for the birth of their first infant, aged between 6 weeks and 12 months old. Participants were recruited through social media platforms via third-sector organisations, namely Dad Matters; a Home-Start project and The Birth Trauma Association. A total of 312 non-birthing parents provided demographic details and obstetric details of the mother’s birth. They also completed questionnaires about their experiences of the birth they were present for, CB-PTSD symptoms, and levels of warmth and invasion in the parent-infant relationship.

**Results:**

Within this sample, 49% experienced the birth they were present for as potentially traumatic. Moreover, 10.1% met clinical criteria for CB-PTSD symptoms, and an additional 7% met sub-clinical criteria. Non-birthing parents who experienced birth as potentially traumatic reported significantly higher CB-PTSD symptoms and felt a greater sense of invasion in relation to their infant. However, levels of warmth in the parent-infant relationship were not statistically different between the two groups. CB-PTSD symptoms had significant associations with invasion but not with warmth, and they mediated the relationship between possible birth trauma and invasion in the parent-infant relationship.

**Conclusions:**

This study’s sample revealed a substantial proportion of non-birthing parents experiencing birth as potentially traumatic, with 10.1% meeting CB-PTSD criteria, a higher incidence than previously reported in the literature. This may be attributed the implications of the COVID-19 pandemic. CB-PTSD symptoms were negatively associated with feelings of invasion in the parent-infant relationship, but not with warmth. Future research should aim to replicate this study design with routine samples of non-birthing parents recruited from maternity settings.

## Background

In the United Kingdom (UK), 91% of fathers^1^ attend the birth of their first child, and 88% attend subsequent births [1]. It is now widely recognised that birth experiences can be perceived as traumatic for some mothers^2^ and non-birthing parents, with growing focus on understanding the psychological impact of such an event [2, 3].

Non-birthing parent involvement during labour has been found to support maternal well-being and reduces the risk of the mother developing childbirth-related post-traumatic stress disorder (CB-PTSD) after a traumatic birth experience [4–6]. However, qualitative research suggests that non-birthing parents often feel excluded from antenatal preparation and unsupported during birth [2, 7–15].

### Traumatic birth experience

Research suggests that up to one in three women appraise their childbirth experience as traumatic [16–18] with an estimated 4.7% prevalance of CB-PTSD [19].

According to the International Classification of Diseases (ICD-11), PTSD requires exposure to “an extremely threatening or horrific event or series of events” [20] in addition to symptoms of re-experiencing, avoidance, and heightened threat perception. These symptoms must have been present for at least several weeks and cause significant impairment in functioning [20].

Although studies on CB-PTSD in women are extensive, studies exploring the impact on non-birthing parents is limited. Bradley and Slade [21] found no significant CB-PTSD symptoms in a sample of 199 non-birthing parents 72 h after they attended the birth of their infant. Helle et al. [22] found CB-PTSD in 1.4% of fathers whose infants were born at very low birth weight (< 1500 g), but none in fathers of infants born at term with no concerns regarding birth weight. Parfitt and Ayers [23] reported 3.8% CB-PTSD prevalence 1–24 months postpartum in fathers while Ayers et al. [24] reported CB-PTSD symptoms in 5% of men 6–12 weeks after birth. Schobinger et al. [25] reported 7.2% prevalence of CB-PTSD in fathers one month after the birth of their infant. A recent meta-analysis by Heyne et al. [19] estimated CB-PTSD prevalence in non-birthing parents at 1.2%, with higher rates in routine samples (2.1%) than in targeted samples (0.8%) where births involved complications, the infant had an admission to the neonatal intensive care unit, their partner delivered via emergency caesarean, or they had a history of trauma. The authors note that the sample size is small (*N =* 562 from 4 studies) and therefore must be interpreted with caution. More research with larger samples is needed to explore prevalence rates of CB-PTSD in non-birthing parents.

### COVID-19

During the Coronavirus disease (COVID-19) pandemic, non-birthing parents’ experiences of maternity care in the UK changed significantly due to varying levels of restricted access to maternity units pre-birth and post-birth [26]. Research suggests that non-birthing parents felt these restrictions negatively impacted their mental health, the couple relationship, social recognition as a parent, and bonding with their infant [27]. Other studies found similar results in that non-birthing parents reported feeling helpless to support their partner giving birth and excluded from the process [28–31]. A meta-analysis of 58 studies highlighted that lack of information regarding their partner and unborn infant was experienced as unpleasant, nerve-wrecking, traumatising, and lonely, and negatively impacted their mental health [32]. In addition, partners felt detached from the birth experience, impairing their ability to bond with their infant [32]. Inconsistent policies across maternity services contributed to confusion, uncertainty, and a perceived reduction in the quality of maternity care [29]. Research in birthing parents found COVID-19 was associated with traumatic childbirth experiences [33]. In Switzerland, researchers found 21.1% of their participants experienced a traumatic childbirth during COVID-19 and 9.1% went on to develop CB-PTSD [34]. In Turkey, the prevalence of CBT-PTSD in birthing parents during COVID-19 was 14.9% [35]. While the impact on the incidence of perceived birth trauma or CB-PTSD prevalence amongst non-birthing parents is still unreported, qualitative evidence suggests that COVID-19 restrictions had a negative effect on non-birthing parents’ experiences of maternity care.

### Impact on the parent-infant relationship

Currently evidence on the impact of CB-PTSD on the parent-infant relationship in non-birthing parents is limited. A key element of parent-infant relationship is the parent’s mental representation of the infant’s feelings towards them [36]. This mental representation is shaped by how the parents perceive their infant’s thoughts, feelings, behaviours, and intentions towards them [37]. This internal representation influences how the parent behaves and interacts with the infant, and therefore has consequences for infant development [36, 37].

CB-PTSD can negatively influence a mother’s ability to form a positive internal representation of their infant [38]. Davies et al. [39] found that, compared to mothers without CB-PTSD, those meeting full or partial CB-PTSD criteria at 6 weeks postpartum viewed their infant less positively, describing them as less warm, more intrusive and demanding, more difficult in temperament, and described their relationship with their infants to be less optimal [39]. Similarly, McDonald et al. [40] found that CB-PTSD at 6 weeks and 3 months postpartum correlated with viewing infants as more demanding at 2 years postpartum, although it did not impact perceptions of infant warmth.

Parfitt and Ayers [23] conducted the only study that has explored the impact of CB-PTSD on the parent-infant relationship in men/fathers. They reported that fathers with CB-PTSD reported a significantly poorer relationship with their infant, although the effect size was small. Given the limited sample size (*N =* 26) further research with larger samples is needed.

In summary, there is growing evidence that non-birthing parents can experience CB-PTSD symptoms from witnessing childbirth. However, the possible impact of perceived birth trauma and CB-PTSD on the parent-infant relationship in non-birthing parents remains unknown, a gap which the current study aims to address.

### Aims

The current study aims to explore the relationships between non-birthing parents’ experiences of childbirth, CB-PTSD symptoms, and their relationship with their infant. It is hypothesised that: (1) Non-birthing parents who experience birth as potentially traumatic will show significantly higher CB-PTSD symptoms, (2) Non-birthing parents who experience birth as potentially traumatic will show significantly higher levels of invasion and lower levels of warmth, (3) Higher levels of CB-PTSD symptoms will be associated with lower levels of warmth and higher levels of invasion in the parent-infant relationship, (4) CB-PTSD symptoms will mediate the relationship between potential traumatic birth experience and the parent-infant relationship. Using CB-PTSD symptoms as a mediator allows the exploration of possible pathways which potential traumatic birth experiences may indirectly affect the parent-infant relationship.

## Methods

### Ethical approval and considerations

Ethical approval was obtained from the University of Liverpool ethics board (Study Reference: 8425).

### Study design

The study used a cross-sectional design.

### Setting

Participants were recruited from community settings in the UK via local birth-trauma organisations and social media. Recruitment took place between January 2021 and February 2022. It is important to note, that during this time, the UK were in varying levels of restriction due to COVID-19, which impacted on maternity services and non-birthing parents’ access to the maternity unit.

### Procedure

This study was a cross-sectional online survey. The online survey was created and administered using Qualtrics, and participants were presented with a participant information sheet and a consent form before beginning the study. Informed consent was obtained from all participants before they could begin the survey. Participants had the right to withdraw consent until the final submission of the questionnaires. Participants were presented with a debrief at the end of the study and if they opted to withdraw at any point during the study, which contained signposting to support services. Participants were recruited via social media through Dad Matters; a Home-Start project and The Birth Trauma Association. Dad Matters; a Home-Start project is a third-sector organisation that provides support to new and expectant fathers across the UK. They aim to provide support with attachment, mental health, and access to services, in partnership with maternity, health visiting and early year support services. The Birth Trauma Association is a third-sector organisation that provides support to parents who have experienced birth as traumatic. They also campaign to increase awareness of birth trauma and signpost parents affected by birth trauma to support services. Both organisations shared information regarding the study via their social media platforms. The majority of recruitment occurred online due to the COVID-19 pandemic restrictions.

### Participants

Participants were non-birthing parents who were present for the birth of their first infant. All non-birth parents were eligible to take part, regardless of their gender identity. The inclusion criteria were that participants were aged 18 + and currently living with the mother and infant. The birth must have been for a single infant at full term (≥ 37 weeks). The birth must have been the first for the woman and the first birth the non-birthing parent had attended. This was to capture the experiences of first-time non-birthing parents with no prior experience of birth. The infant must have been aged between 6 weeks and 12 months old at the time of completing the study. Participants were required to have access to an internet-connected device and to be able to read and understand English to complete measures. Exclusion criteria were non-birthing parents currently receiving care from a psychiatrist, the rationale being that those under the care of a psychiatrist are likely to be experiencing pre-existing severe mental health difficulties which might themselves impact on the relationship with the infant. Other exclusion criteria were partners of mothers who remained in the hospital for more than seven days following birth, and whose infant spent time in neonatal intensive care, or more than 72 h in the special care baby unit, the rationale being that this early separation may have influenced relationship building in the early postnatal period. These exclusion criteria must be considered when interpreting the findings in terms of generalizability.

### Variables

#### Demographic and obstetric information

Demographic information relating to the participant and obstetric information relating to the mother’s pregnancy and birth were collected via the non-birthing parent.

#### Experience of birth as potentially traumatic

To ascertain whether the birth was experienced as potentially traumatic, participants were asked ‘Thinking about the childbirth that you witnessed, was there any time during this when you felt: (1) horror or helplessness about what was happening? (2) really frightened about your partner’s or your infant’s wellbeing?’. These questions were developed in the context of a previous study with experts by experience at the Birth Trauma Association [41] and are in line with the ICD-11, which states that for PTSD to be present, there must have been exposure to an event/s of an “extremely threatening *or* horrific nature” [20]. These questions capture the non-birthing parents’ appraisal of the perceived threats and their emotional responses during the birth. In women, their subjective appraisal during the birth experience is a risk factor for developing CB-PTSD, and therefore, the questions were adapted for use with non-birthing parents in the current study to ensure that appraisal and emotional responses were captured [41–44]. A birth was deemed to have been experienced as potentially traumatic if the participant answered yes to both questions.

#### CB-PTSD symptoms

CB-PTSD symptoms were measured using the Impact of Event Scale-Revised (IES-R) [45]. The IES-R is a 22-item self-report measure of PTSD symptoms. Items are rated from 0 (‘not at all’) to 4 (‘extremely’) concerning how distressing each item has been during the past week about a specific event. The event was specified as the labour and birth and immediately after. The scale has three subscales: intrusion (‘Any reminder of the birth brought back feelings about it’; 8 items; score range 0–32), avoidance (‘I stayed away from reminders of the birth’; 8 items; score range 0–32), and hyperarousal (Reminders of the birth caused me to have physical reactions, such as sweating, trouble breathing, nausea, or a pounding hear’; 6 items; score range 0–24). Higher scores (range 0–88) indicate higher PTSD symptoms. A total score of 24 and above indicates that PTSD is a clinical concern, and a total score of 33 and above is considered to signify the likely presence of PTSD symptoms [45–47]. In this study, the scale had a Cronbach’s alpha value of 0.95, indicating excellent internal consistency between items. The IES-R has been used in previous studies looking at CB-PTSD in non-birthing parents [5, 19, 22].

#### The parent-infant relationship

The parent-infant relationship was measured using the Mothers’ Object Relations Scale-Short Form (MORS-SF) [36]. The MORS-SF is a 14-item self-report screening tool used to identify potential areas of difficulty in the parent-infant relationship. Items are rated from 0 (‘never’) to 5 (‘Always’). The scale has two subscales: warmth (‘My baby smiles at me’; 7 items; score range 0–35) and invasion (‘My baby wants too much attention’; 7 items; score range 0–35). Warmth refers to how the parent perceives the child’s feelings towards them through factors such as smiling, laughing, and showing affection. Higher scores indicate higher perceived warmth in the parent-infant relationship. A warmth score below 20 may indicate concern, and a score of 11 or less should indicate concern [36]. Invasion refers to how dominating or intrusive the parent perceives the child to be. Higher scores indicate higher perceived invasion in the parent-infant relationship. An invasion score higher than 12 may indicate possible concern, and a score of 17 and above should indicate concern [36]. The measure is validated for use with mothers and fathers of children aged 6 weeks to 12 months old. In this study, the MORS-SF warmth subscale had a Cronbach’s alpha value of 0.87 and the invasion subscale had a Cronbach’s alpha value of 0.82, both indicating good internal consistency between items.

### Study size

The study sample size was calculated using ‘GPower 3.1’ software based on two-tailed independent t-test analysis with a medium effect size of 0.5, an alpha of 0.05, and power of 0.8 yielding a final sample size of at least 128 participants.

### Analysis

The Statistical Package for the Social Sciences (SPSS) version 27 was used for data analysis. Data were assessed for normality. Correlation, t-test, and Analysis of Variance (ANOVA) analyses were used to identify potential confounding variables on potential traumatic birth experience, CB-PTSD symptoms, and the parent-infant relationship.

To test hypotheses one and two, a one-way Analysis of Covariance (ANCOVA) was used to compare PTSD symptoms across the birth experienced as potentially traumatic compared to the birth not experienced as potentially traumatic, controlling for identified confounding variables.

Hypothesis three was tested using hierarchical regression to assess the relationship between CB-PTSD symptoms and warmth and invasion in the parent-infant relationship.

Hypothesis four was tested using mediation analysis via the PROCESS macro and the Hayes & Preacher approach [48] to determine the indirect effects of CB-PTSD symptoms on the relationship between potentially traumatic birth experience and parent-infant relationship. Bias-corrected and accelerated bootstrapping (5000 bootstrap resamples) were used to generate the confidence interval (CI) around the indirect effect estimate [49].

## Results

Data were assessed for normality and parametric tests were utilised for analyses. Where data was missing, this is represented by Ns in the relevant tables.

### Participants

The final sample was 312. Ages ranged from 21 to 44 years (*M* = 32.6, *SD* = 4.5). The age of the participants’ infants ranged from 6 weeks to 12 months (*M* = 164.2 days, *SD* = 91). The gender and marital status of the participants can be seen in Table 1. Most of the sample identified as male (99%), and over half were married (60.9%). Obstetric information from the mother’s birth experience can be seen in Table 2. Nearly half the births had no intervention (45.2%). Mean scores on the IES-R and MORS-SF comparing where the birth was or was not experienced as potentially traumatic can be seen in Table 3. Most of the sample (*N* = 297, 95.2%) were recruited outside of COVID-19 pandemic restrictions and only 34% (*N* = 106) had infants born during restrictions.

**Table 1:** Sample demographics

Demographic variable	Overall sample (*N* = 312) *N* (%)	Experience of birth as not traumatic (*N* = 158) *N* (%)	Experience of birth as potentially traumatic (*N* = 152) *N* (%)
Gender			
Male	309 (99.0)	157 (99.4)	151 (99.3)
Female	1 (0.3)	0 (0.0)	0 (0.0)
Non-binary	1 (0.3)	1 (0.6)	0 (0.0)
Transgender male	1 (0.3)	0 (0.0)	1 (0.7)
Marital status			
Single/non-partnered	102 (32.9)	52 (32.9)	50 (32.9)
Married	188 (60.6)	94 (59.5)	94 (61.8)
Registered partnership	20 (6.5)	12 (7.6)	8 (5.3)

**Table 2:** Obstetric demographics

Obstetric variable	Overall sample (*N* = 312) *N* (%)	Experience of birth as not traumatic (*N* = 158) *N* (%)	Experience of birth as potentially traumatic (*N* = 152) *N* (%)
Was induction required? ^a^			
Yes	143 (46.1)	60 (38.0)	82 (53.9)
No	167 (53.9)	98 (62.0)	68 (44.7)
Mode of delivery			
No intervention	141 (45.2)	79 (50.0)	62 (40.8)
Planned caesarean	41 (13.1)	28 (17.7)	13 (8.6)
Assisted vaginal delivery	52 (16.7)	21 (13.3)	31 (20.4)
Emergency caesarean	78 (25.0)	30 (20.0)	46 (30.2)
Complications during pregnancy? ^a^			
Yes	56 (18.1)	24 (15.2)	32 (21.3)
No	254 (81.9)	134 (84.8)	118 (78.7)
Complications during labour/birth?			
Yes	86 (27.6)	21 (13.3)	65 (42.8)
No	226 (72.4)	137 (86.7)	87 (57.2)

Note: ^a^Not all questions were completed by all participants. As such, valid percentages have been reported

**Table 3:** PTSD and parent-infant relationship scores

Variable	Overall sample (*N* = 310) M (SD)	Experience of birth as not traumatic (*N* = 141) M (SD)	Experience of birth as potentially traumatic (*N* = 146) M (SD)
IES-R			
Intrusions	4.38 (5.59)	2.45 (3.77)	6.40 (6.44)
Avoidance	4.17 (5.58)	1.89 (3.14)	6.44 (6.48)
Hyperarousal	2.16 (3.61)	1.37 (2.71)	3.02 (4.23)
Total	10.87 (13.81)	5.71 (8.80)	15.86 (15.81)
MORS-SF			
Warmth	24.08 (6.12)	23.93 (6.46)	24.23 (5.80)
Invasion	10.26 (5.39)	9.44 (4.95)	11.11 (5.71)

Note: M = mean; SD = standard deviation

Not all questions were completed by all participants. As such, valid percentages have been reported

### Experience of birth as potentially traumatic

This study found that 49% (*N* = 152) of the sample had experienced birth as potentially traumatic. 60% (*N* = 186) reported feeling horror or helplessness during the birth, and 63.2% (*N* = 196) reported feeling frightened about their partner’s or their infant’s wellbeing during the birth.

### Prevalence of CB-PTSD

Completed scores ranged from 0 to 32 on the intrusion subscale (*M* = 4.38, *SD =* 5.59), 0–32 on the avoidance subscale (*M* = 4.17, *SD =* 5.58), and 0–24 on the hyperarousal subscale (*M* = 2.16, *SD =* 3.61). The total score on the IES-R ranged from 0 to 88 (*M* = 10.87, *SD =* 13.81). Descriptive statistics of CB-PTSD symptoms can be seen in Table 4. Of note, 10.1% (*n* = 29) met the criteria for fully symptomatic CB-PTSD and 7% (*N* = 20) met the criteria for subclinical CB-PTSD symptomology. Table 3 shows the mean IES-R score for the birth experienced as potentially traumatic group (*M =* 13.05, *SD =* 14.58) was still much lower than the clinical cut-off indicative of CB-PTSD symptoms (score ≥ 33).

**Table 4:** Prevalence of clinical levels of PTSD symptoms and warmth and invasion

Variable	Overall sample *N* (%)	Experience of birth as not traumatic *N* (%)	Experience of birth as potentially traumatic *N* (%)
IES-R			
No PTSD (Score 0–23)	238 (82.9)	133 (94.3)	105 (71.9)
Partial PTSD (Score 24–32)	20 (7.0)	4 (2.8)	16 (11.0)
Fully symptomatic PTSD (Scores ≥ 33)	29 (10.1)	4 (2.8)	25 (17.1)
MORS-SF			
Warmth no concern (Score 21–35)	223 (74.3)	109 (72.7)	114 (76.0)
Lack of warmth possible concern (Score 12–20)	68 (22.7)	36 (24.0)	32 (21.3)
Lack of warmth definite concern (Score 0–11)	9 (3.0)	5 (3.3)	4 (2.7)
Invasion no concern (Score 0–11)	190 (63.1)	105 (70.0)	85 (56.3)
Invasion possible concern (Score 12–16)	70 (23.3)	31 (20.7)	39 (25.8)
Invasion definite concern (Score 17–35)	41 (13.6)	14 (9.3)	27 (17.9)

Note: Not all questions were completed by all participants. As such, valid percentages have been reported

### Parent-infant relationship

Scores on the warmth subscale ranged from 1 to 35 (*M* = 24.08, *SD =* 6.12) and 0–28 on the invasion subscale (*M* = 10.26, *SD* = 5.39). In the current sample, lack of warmth was a possible concern (score 12–20) for 22.7% (*N* = 68) and a definite concern (score 0–11) for 3% (*N* = 9). Invasion was a possible concern (score 12–16) for 23.3% (*N* = 70) of the sample and a definite concern (score 17–35) for 13.6% (*N* = 41). Table 4 shows MORS-SF scores for the overall sample and according to whether the birth was experienced as potentially traumatic or not.

### Confounders

Correlation, t-test, and ANOVA analyses were completed to identify potential confounding variables to control for in subsequent analyses. Results are presented in Table 5. Participant age was significantly negatively correlated with intrusions (*r*(268) = − 0.14, *p* = .025). CB-PTSD symptoms were significantly higher in those who had a partner who was perceived to have experienced complications during pregnancy (e.g. hyperemesis gravidarum, gestational diabetes, preeclampsia) (*M* = 16.54, *SD* = 16.13) than in those who did not (*M* = 9.46, *SD* = 12.69) (*t*(65.79) = -2.97, *p* = .004, *d* = − 0.53). Participant age and complications during pregnancy were therefore controlled for in subsequent analyses. Infant age was significantly correlated with warmth (*r*(300) = 0.40, *p* <. 001) and was therefore controlled for in further analyses concerning warmth. Marital status, whether the pregnancy was planned, mode of delivery, and whether induction was required were not associated with CB-PTSD symptoms or the parent-infant relationship.

**Table 5:** Confounding variable analyses

Variable	IES-*R* Intrusions	IES-*R* Avoidance	IES-*R* Hyperarousal	IES-*R* Total	MORS-SF Warmth	MORS-SF Invasion
Infant age (*r*)	-0.06	-0.06	-0.08	-0.07	0.40**	0.08
Parent age (*r*)	-0.14**	-0.06	-0.07	-0.10	-0.08	-0.07
Marital status (*F*)	1.18	0.57	1.40	0.83	0.99	0.37
Planned/unplanned pregnancy (*t*)	1.57	0.47	0.66	1.37	0.30	-1.21
Mode of delivery (*F*)	1.35	0.86	0.23	0.98	1.26	1.78
Was induction required (*t*)	0.91	0.72	1.24	0.92	0.29	-0.24
Complications during pregnancy (*t*)	-2.61**	-2.95**	-2.42**	-2.97**	-0.64	-1.73

Note: ** indicated p significant at the 0.05 value. *** indicates *p* < .001

### Hypothesis 1: non-birthing parents who experience birth as potentially traumatic will show significantly higher PTSD symptoms

The results indicated that non-birthing parents who experienced the birth as potentially traumatic reported significantly higher CB-PTSD symptoms than non-birthing parents who did not, with a medium effect size (*F*(1, 255) = 23.22, *p* < .001, η^2^ = 0.083). This was the case for all subscales: intrusions (*F*(1, 262) = 24.95, *p* < .001, η^2^ = 0.087), avoidance (*F*(1, 262) = 25.22, *p* < .001, η^2^ = 0.088), and hyperarousal (*F*(1, 265) = 8.52, *p* = .004, η^2^ = 0.031). Means and standard deviations are displayed in Table 3.

### Hypothesis 2: non-birthing parents who experience birth as potentially traumatic will show significantly higher levels of invasion and lower levels of warmth

While no significant effect of potentially traumatic birth experience was found on warmth in the parent-infant relationship (*F*(1, 298) = 0.18, *p* = .67, η^2^ = 0.00), the levels of invasion were found to be significantly higher in non-birthing parents who found birth to be potentially traumatic compared to those who did not, with a medium effect size (*F*(1, 299) = 6.75, *p* = .010, η^2^ = 0.02). Means and standard deviations are displayed in Table 3.

### Hypothesis 3: higher levels of CB-PTSD symptoms will be associated with lower levels of warmth and higher levels of invasion and in the parent-infant relationship

Assumptions for regression were met, and model diagnostics did not raise any concerns. Concerning warmth, infant age was entered into the model as step 1, and the IES-R total score was entered as step 2. Table 6 shows the regression coefficients. Results indicated no significant relationship between CB-PTSD symptoms and warmth.

Concerning invasion, participant age was entered into the model as step 1, complications during pregnancy as step 2, and IES-R total score entered as step 3. Table 7 shows the regression coefficients. CB-PTSD was significantly associated with invasion. Overall, the regression model accounted for 5.6% of the variance in invasion, with CB-PTSD symptoms accounting for 4.1% of the variance in invasion. Participant age and complications during pregnancy were not significantly associated with invasion, accounting for 0.4% and 1.5% of the variance, respectively.

**Table 6:** Regression results with warmth as the dependent variable

Variable	b	β	95% CI for b [LB, UB]	*p*	Fit	Difference
Intercept	19.59***		[18.24, 20.95]	< 0.001***		
Infant age	0.03***	0.40	[0.02, 0.03]	< 0.001***		
					*R*^*2*^ *=* 0.163***	
Intercept	19.63***		[18.14. 21.12]	< 0.001***		
Infant age	0.03***	0.40	[0.02, 0.03]	< 0.001***		
IES-R total	-0.003	-0.01	[-0.05, 0.05]	0.91		
					*R*^*2*^ *=* 0.163***	*R*^*2*^*change* = 0.001

Note: B = unstandardised coefficient; SE = standard error; β = standardised coefficient; CI = confidence interval; LB = lower bound; UB = upper bound; *** indicates *p* < .001

**Table 7:** Regression results with invasion as the dependent variable

Variable	b	β	95% CI for b [LB, UB]	*p*	Fit	Difference
Intercept	12.66***		[7.88, 17.44]	< 0.001***		
Age	-0.08	-0.06	[-0.22, 0.07]	0.31		
					*R*^*2*^ *=* 0.004	
Intercept	10.94***		[5.76, 16.12]	< 0.001***		
Age	-0.08	-0.06	[-0.22, 0.07]	0.31		
Complications during pregnancy	1.44	0.10	[-0.26, 3.15]	0.10		
					*R*^*2*^ *=* 0.015	*R*^2^ change = 0.011
Intercept	9.93***		[4.81, 15.04]	< 0.001***		
Age	-0.05	-0.04	[-0.19, 0.09]	0.50		
Complications during pregnancy	0.84	0.06	[-0.88, 2.55]	0.39		
IES-R Total	0.08***	0.21	[0.03, 0.13]	< 0.001***		
					*R*^*2*^ *=* 0.056***	*R*^*2*^*change* = 0.041**

Note: b = unstandardised beta; SE = standard error; β = standardised beta; CI = confidence interval; LB = lower bound; UB = upper bound; ** indicates *p* < .01; *** indicates *p* < .001

### Hypothesis 4: CB-PTSD symptoms will mediate the relationship between potentially traumatic childbirth experience and the parent-infant relationship

**Fig. 1:**
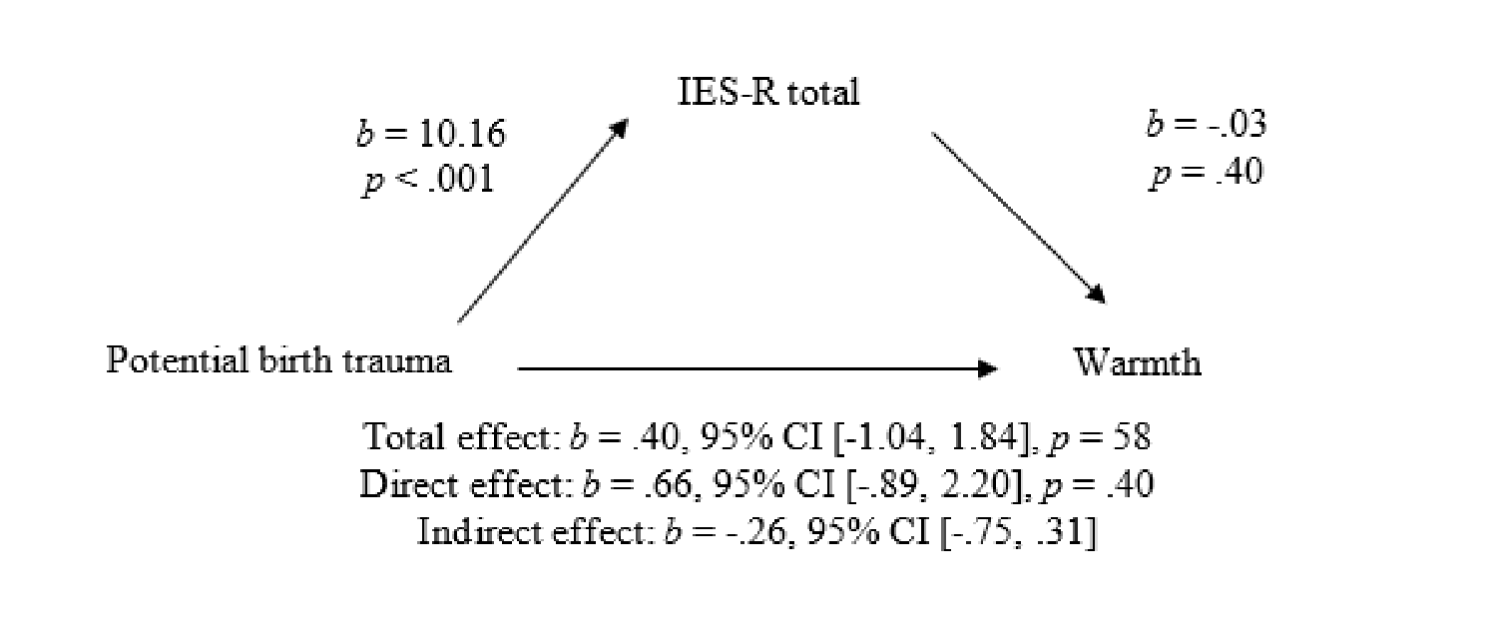
Mediation model of the relationship between potentially traumatic birth experience and warmth, mediated by CB-PTSD symptoms. The confidence interval (CI) for the indirect effect is a bootstrapped CI Based on 5000 Samples

Mediation was conducted for potentially traumatic birth experience and warmth and invasion using bootstrapping with 5000 samples. Figure 1 shows the mediation model for potentially traumatic birth experience and warmth, with CB-PTSD as the mediator variable. The results showed that there was no relationship between traumatic birth experience and warmth and that CB-PTSD was not a mediating variable.

**Fig. 2:**
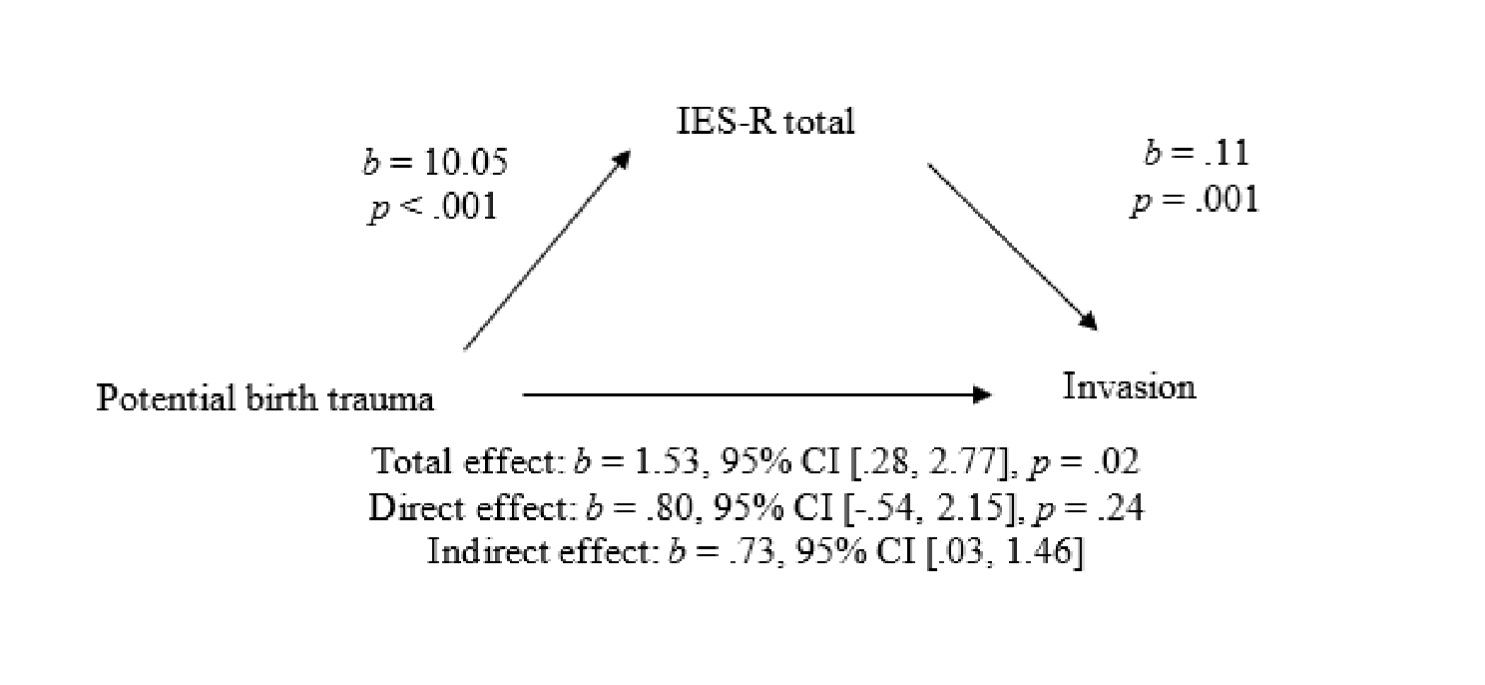
Mediation model of the relationship between potentially traumatic birth experience and invasion, mediated by CB-PTSD symptoms. The confidence interval (CI) for the indirect effect is a bootstrapped CI Based on 5000 Samples

Figure 2 shows the mediation model for potentially traumatic birth experience and invasion. The results showed that the relationship between potentially traumatic birth experience and invasion, without the mediator in the model, was significant. When CB-PTSD symptoms were added to the model as the mediator, the total effect of potentially traumatic birth experience on invasion became non-significant. The mediation analyses indicated a significant indirect effect of CB-PTSD symptoms, which mediated the relationship between potentially traumatic birth experience and perception of the infant as invasive.

## Discussion

The study aimed to explore the relationships between non-birthing parents’ experiences of childbirth, CB-PTSD symptoms, and the non-birthing parent’s relationship with their infant.

Firstly, as hypothesised, non-birthing parents who perceived the birth as potentially traumatic showed significantly higher CB-PTSD symptoms compared to those who did not. However, their mean CB-PTSD score remained well below the clinical threshold (score ≥ 33), indicating that while symptoms were elevated in this group, they did not reach levels of clinical concern This aligns with findings from Helle et al. [22], where fathers had a mean CB-PTSD (IES-R) score of 9.8 (*SD* = 7.8), lower than in the current study, but still below the clinical cut-off.

Secondly, the hypothesis concerning warmth in the parent-infant relationship was not supported. Non-birthing parents who experienced birth as potentially traumatic did not perceive lower levels of warmth in the relationship with their infant. As the first study to explore this association in non-birthing parents, these findings suggest that potentially traumatic childbirth experiences may not affect perceived warmth in the relationship, aligning with previous research by McDonald et al. [40] who found no significant association between CB-PTSD and warmth in mothers.

The hypothesis regarding invasion were supported. Non-birthing parents who perceived the birth as potentially traumatic reported significantly higher invasion scores, indicating that they were more likely to interpret their infants’ behaviours as intrusive and demanding. Again, this is the first study to examine this association in non-birthing parents, but the findings are aligned with previous research in mothers by McDonald et al. [40] who found a small but significant correlation between CB-PTSD and invasion.

In the current sample, 22% reported a lack of warmth that was a possible concern and 3% had a lack of warmth reaching clinically concerning levels. This means that a quarter of the sample showed some level of concern regarding warmth in their internal representation of their infant. For invasion, 22% had invasion levels that were a possible concern and 13% had levels reached clinically concerning levels, indicating that over a third of the sample had heightened invasion scores. The mean invasion scores in the current sample were higher than those in Davies et al. [39], who examined the impact of CB-PTSD on the mother-infant relationship in a routine sample recruited via a large maternity hospital. By contrast, the current sample was primarily recruited from social media via third sector organisations providing mental health support to non-birthing parents, which may explain the elevated invasion scores due to potential sampling bias.

Regarding hypothesis three, CB-PTSD symptoms was not associated with lower levels of warmth, suggesting that these symptoms did not influence perceptions of warmth from the infant. However, as hypothesised, CB-PTSD symptoms were significantly associated with invasion, indicating that non-birthing parents with CB-PTSD symptoms may interpret their infant’s behaviour towards them as more intrusive and demanding. This aligns with Parfitt and Ayers [23], who found that non-birthing parents with CB-PTSD symptoms reported a significantly more negative relationship with their infant. Their sample size was small (*N =* 26), and more research with larger, more representative samples from routine maternity settings is needed to explore these associations further.

Despite being significant, the regression model accounted for only 5.6% of the variance in invasion, with CB-PTSD symptoms accounting for 4.1% of the variance, suggesting there are other factors not considered in this study that may explain differences in perceptions of invasion. For example, depressive symptoms have been suggested to influence the parent-infant relationship and were not accounted for in this study [23, 39, 40]. McDonald et al. [40] and Davies et al. [39] found that associations between CB-PTSD symptoms and the parent-infant relationship became non-significant once postnatal depressive symptoms were controlled for in mothers. However, the direction is uncertain, as a common consequence of CB-PTSD symptoms is low mood. The absence of concurrent assessment of depressive symptoms is a limitation of the study design and future research could build on this by including a measure of depression.

Finally, CB-PTSD symptoms were found to mediate the relationship between possible birth trauma and invasion, but not warmth. CB-PTSD symptoms such as intrusions, hyperarousal, and avoidance may be particularly challenging to manage when caring for a new infant, who requires continuous care and attention. Consequently, non-birthing parents experiencing such symptoms may perceive their infant as behaving more difficult and intrusive towards them. This is the first study to explore this mediating relationship in non-birthing parents and adds a novel contribution to the literature. Future research should build on this by exploring this mediating relationship in routine samples alongside use of a clinical interview to identify CB-PTSD.

The recruitment period for this study included varying levels of COVID-19 restrictions in the UK. These changes may have influenced how participants felt about the birth experience and their mental health. In addition, the pandemic may have influenced non-birthing parents’ mental health, a deterioration of which may have altered their perception of the birth or predisposed them to CB-PTSD symptoms. Research suggests that during COVID-19, non-birthing parents felt that the restrictions in maternity services negatively impacted their mental health, the couple relationship, social recognition as a parent, and bonding with their infant [27, 32], which may explain the high incidence of possible birth trauma and CB-PTSD in the current sample. However, as most of the sample were recruited outside of pandemic restrictions, the authors believe the implications of COVID-19 in this study were limited. In addition, the sampling bias may further explain the high incidence of possible birth trauma and CB-PTSD. It is therefore important for further research to build on this study by exploring the associations in routine samples of non-birthing parents, recruited following the end of the COVID-19 pandemic.

### Limitations

It must be noted that the focus of this study is on symptoms and not on diagnosed CB-PTSD. In addition, the cross-sectional design of the current study is a limitation. The study design precludes causal inferences, and the hypothesis testing does not consider the potential impact of the parent-infant relationship on CB-PTSD symptoms and the perception of the birth experience. Future research could build on this by utilising a longitudinal design, collecting data on non-birthing parents’ mental health during their partners pregnancy through to the postnatal period. In addition, use of clinical interview would support the assessment and diagnosis of CB-PTSD.

The conceptualisation of birth trauma in the current sample may have resulted in a high incidence of reported perceived birth trauma. Half the current sample found the birth that they were present for to be a potentially traumatic experience. Rates for exposure to potentially traumatic birth in non-birthing parents have not been reported in prior studies and therefore it is not possible to make comparisons; however, it appears probable that this is a sample with a high rate of possible trauma exposure. The rates of CB-PTSD symptoms in the current sample were higher than those currently reported in the literature, with 10.1% meeting criteria for clinical symptoms of CB-PTSD and an additional 7% displaying subclinical symptomology. This is much higher than the rate of 1.2% reported in a recent meta-synthesis of CB-PTSD in non-birthing parents [19]. The high trauma incidence may be due to sampling bias and possibly a result of the context of COVID-19. To obtain a more representative understanding, future research should aim to recruit routine consecutive samples of non-birthing parents and explore prevalence rates of perceived birth trauma and CB-PTSD symptomology. In addition, the use of a clinical interview alongside self-report measures may also offer better insights into prevalence rates.

The sample had a wide range of infant ages. Parents’ internal working models of their infants are likely to undergo changes during the first year of the infant’s life. However, it may also be expected that some elements of the internal working model of their infant will remain similar, in terms of enduring perceptions of more negative or positive representations of their infant’s feelings towards them [36]. Age-to-age stability was assessed in the original validation of the MORS-SF [36] and the subscales of warmth and invasion were found to have good age-to-age stability from 6-months of age to 12-months. Such a wide range of infant age in the current sample, captured with cross-sectional design, limits the ability to make inferences regarding what may be stable versus what may have been subject to age-related change in the parent-infant relationship. Future research should seek to build on this by utilising a longitudinal design or limiting the age range of infants in the inclusion criteria.

It is important to consider the demographic of the sample in the current study are mostly males who are married and in attendance at the birth of their first infant. Non-birthing parents who were receiving care from a psychiatrist, or had a partner who stayed in hospital for over 7 days are not captured in this study and therefore the findings are not generalizable to these groups. In addition, non-birthing parents who have attended multiple births are also not captured in this sample. More research is needed to explore the experiences of these non-birthing parents. In addition, limited sociodemographic data were collected from participants, to increase ease of study participation, however, this does limit the depth of understanding of the results in terms of generalisability and whether the sample reflects the general population of the UK.

### Clinical implications

The results from this study suggest that CB-PTSD symptoms may be one mechanism by which a relationship between experiencing birth as potentially traumatic and invasion in the parent-infant relationship exists. If findings are replicated by further research, outside of the COVID-19 pandemic context, this may suggest the need to consider screening non-birthing parents for whether they experienced the birth as potentially traumatic.

## Conclusion

To conclude, this study found that a high proportion of non-birthing parents in this sample experienced birth as potentially traumatic (49%) and 10.1% of this sample showed symptoms of CB-PTSD. Non-birthing parents who experienced birth as potentially traumatic displayed higher levels of CB-PTSD symptoms and higher levels of invasion in the parent-infant relationship. Warmth in the parent-infant relationship remained unaffected by potential birth trauma or CB-PTSD symptomology. Furthermore, this research found that CB-PTSD symptoms were found to mediate the relationship between potentially traumatic birth experience and invasion in the parent-infant relationship. The COVID-19 pandemic context is important to consider and may explain the high incidence of potentially traumatic childbirth experiences of CB-PTSD. More research is needed into routine samples of non-birthing parents recruited from maternity settings to explore whether these associations still exist.

## Data Availability

The datasets used and/or analysed during the current study are available from the corresponding author on request.

## Footnotes

1 The term father is used here as this is the terminology used within the cited research specifically. The term non-birthing parents will be used from hereon in to refer to birth partners in a co-parenting role. ^2^ The term mother is used here but the authors recognise that not all female gestational parents will identify with this terminology.

## Abbreviations

ANCOVA: Analysis of Covariance
ANOVA: Analysis of Variance
CB-PTSD: Childbirth-Related Post-Traumatic Stress Disorder
CI: Confidence Interval
COVID-19: Coronavirus
ICD-11: International Classification of Diseases 11th Edition
IES-R: Impact of Event Scale-Revised
M: Mean
MORS-SF: Mothers’ Object Relations Scale-Short Form
PTSD: Post-Traumatic Stress Disorder
SD: Standard Deviation
SPSS: Statistical Package for the Social Sciences
UK: United Kingdom
